# Acute Pain in Right Iliac Fossa during Pregnancy

**DOI:** 10.5334/jbsr.2807

**Published:** 2022-04-26

**Authors:** Ophelye Chiabai, Cristina Anca Dragean

**Affiliations:** 1 UCL, BE

**Keywords:** Epiploic appendagitis, right iliac fossa, pregnancy

## Abstract

**Teaching Point:** The diagnosis of epiploic appendagitis in case of pain in the right iliac fossa in pregnant woman shouldn’t be forgotten.

## Case History

**Figure 1:**
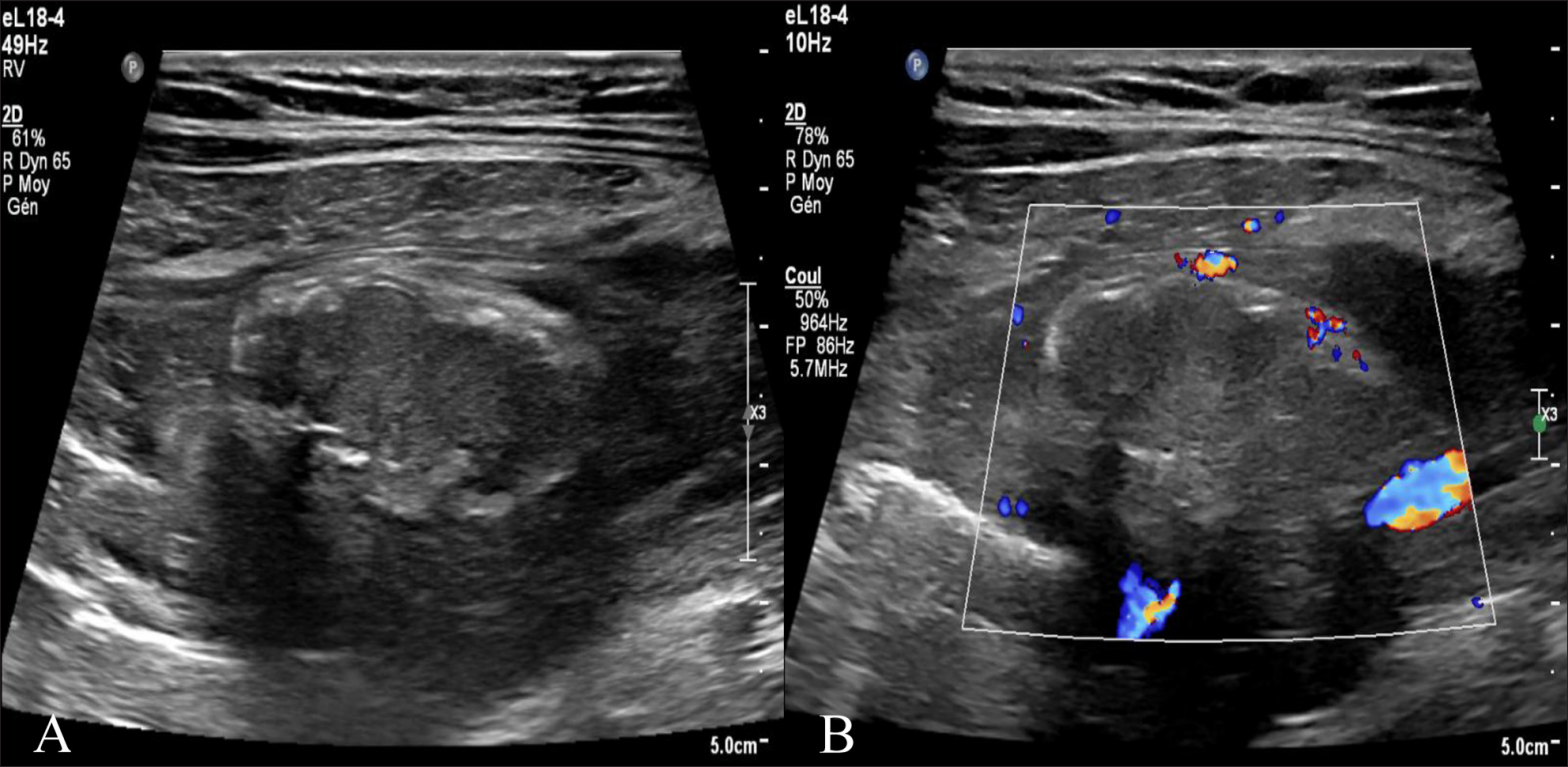
**Right iliac fossa ultrasound. A)** Superficial intraperitoneal oblong mass in the right iliac fossa of 35mm non-compressible, hyperechoic, surrounded by a subtle hypoechoic line. **B)** without internal vascularity.

**Figure 2:**
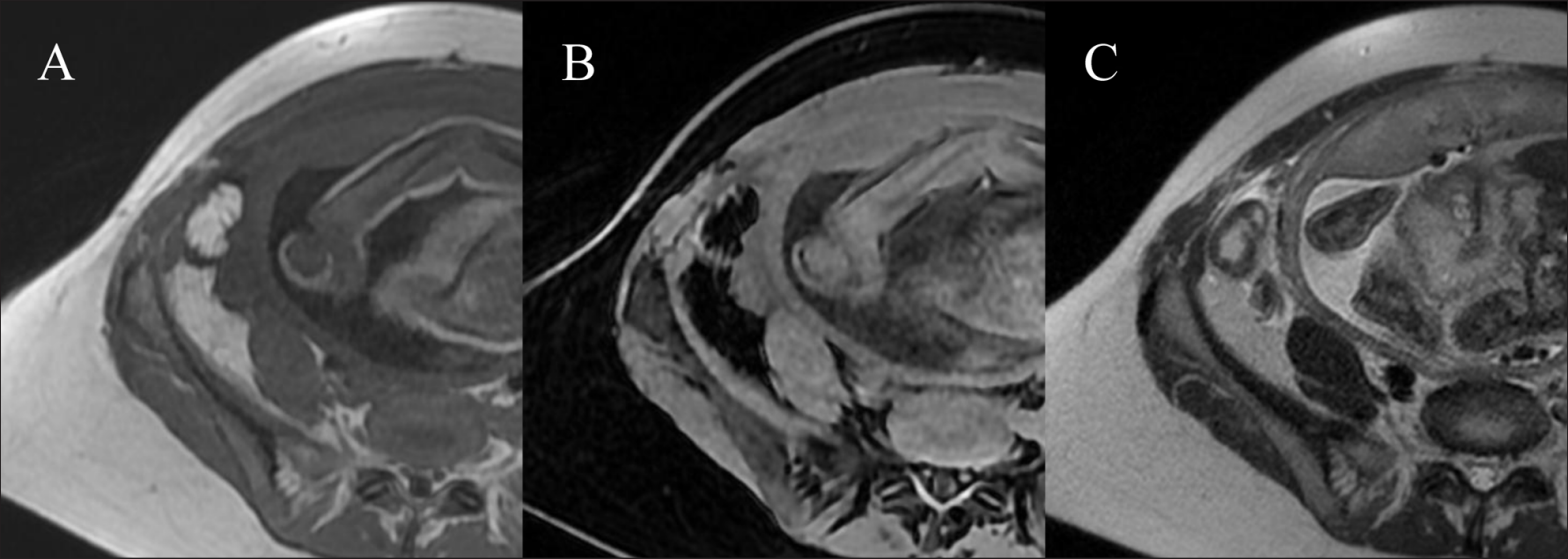
**Right iliac fossa MRI. A)** T1 sequence showed a anterior superficial intraperitoneal oblong mass in the right iliac fossa of 35mm hypersignal slightly less intense than normal fat with a thin peripheral hyposignal. **B)** T1FS sequence showed hyposignal of the mass with a fine periperal hypersignal. **C)** Hypersignal T2 of the mass and hypersignal T2 in the fat around.

A 36-year-old pregnant woman (35W4D) arrived at the emergency room after having pain in the right iliac fossa for two days. Blood tests showed a mild inflammatory syndrome. Urinary tests were negative and the gynecological examination was normal. An abdominal ultrasound was performed to exclude acute appendicitis. This ultrasound showed a normal appendix but also an anterior superficial intraperitoneal oblong mass in the right iliac fossa of 35mm non-compressible, hyperechoic, surrounded by a subtle hypoechoic line (***Figure 1A***) and without internal vascularity (***Figure 1B***) [1]. There was no bowel wall thickening or ascites. The ultrasound (US) diagnosis was epiploic appendagitis. The computed tomography (CT) scanner was not an option in this specific case to confirm this diagnosis. Magnetic resonance imaging (MRI) was performed and confirmed the presence of an anterior superficial intraperitoneal oblong mass in the right iliac fossa of 35mm hypersignal in T1 slightly less intense than normal fat (due to inflammatory stranding) with a thin peripheral hyposignal T1 (***Figure 2A***), hyposignal with a fine periperal hypersignal T1FS (***Figure 2B***), hypersignal T2 (***Figure 2C***).

## Commentary

Acute abdominal pain in pregnancy requires a different imaging approach to avoid exposure to X-rays. Ultrasound remains the first-line modality due to its wide availability and ability to diagnose a range of abdominal diseases. However, due to changes in the location of organs caused by displacement by the gravid uterus, identification of structures can be impossible, and as such MRI has taken on a growing role in this scenario. The differential diagnosis of abdominal pain in the right iliac fossa during pregnancy is quite broad. It must contain the ovarian causes usually explored initially by specialists via endovaginal examination. Then the renal causes which are easily identifiable by ultrasound. Acute appendicitis can also be identified by ultrasound but this approach is more difficult with the ascent of the caecum in the upper right quadrant as the gestational age increases. In this case, the use of MRI without injection of contrast product may be necessary. Right diverticulitis and epiploic appendagitis are more rare than acute appendicitis and should not be discarded.
